# Dynamics and concordance alterations of regional brain function indices in vestibular migraine: a resting-state fMRI study

**DOI:** 10.1186/s10194-023-01705-y

**Published:** 2024-01-05

**Authors:** Xing Xiong, Lingling Dai, Wen Chen, Jiajie Lu, Chunhong Hu, Hongru Zhao, Jun Ke

**Affiliations:** 1 Department of Radiology, The First Affiliated Hospital of Soochow University, Suzhou, 215006 Jiangsu China; 2 Institute of Medical imaging, Soochow University, Soochow, Jiangsu Province People’s Republic of China; 3 Department of Neurology, The First Affiliated Hospital of Soochow University, Suzhou, 215006 Jiangsu China

**Keywords:** Vestibular migraine, Temporal dynamic analysis, Intrinsic brain activity, Resting-state fMRI

## Abstract

**Background:**

Prior MRI studies on vestibular migraine (VM) have revealed abnormalities in static regional intrinsic brain activity (iBA) and dynamic functional connectivity between brain regions or networks. However, the temporal variation and concordance of regional iBA measures remain to be explored.

**Methods:**

57 VM patients during the interictal period were compared to 88 healthy controls (HC) in this resting-state functional magnetic resonance imaging (fMRI) study. The dynamics and concordance of regional iBA indices, including amplitude of low-frequency fluctuations (ALFF) and regional homogeneity (ReHo), were examined by utilizing sliding time-window analysis. Partial correlation analyses were performed between clinical parameters and resting-state fMRI indices in brain areas showing significant group differences.

**Results:**

The VM group showed increased ALFF and ReHo dynamics, as well as increased temporal concordance between ALFF and ReHo in the bilateral paracentral lobule and supplementary motor area relative to the HC group. We also found decreased ReHo dynamics in the right temporal pole, and decreased ALFF dynamics in the right cerebellum posterior lobe, bilateral angular gyrus and middle occipital gyrus (MOG) in the VM group compared with the HC group. Moreover, a positive correlation was observed between ALFF dynamics in the left MOG and vertigo disease duration across all VM patients.

**Conclusion:**

Temporal dynamics and concordance of regional iBA indices were altered in the motor cortex, cerebellum, occipital and temporoparietal cortex, which may contribute to disrupted multisensory processing and vestibular control in patients with VM. ALFF dynamics in the left MOG may be useful biomarker for evaluating vertigo burden in this disorder.

**Supplementary Information:**

The online version contains supplementary material available at 10.1186/s10194-023-01705-y.

## Introduction

Vestibular migraine (VM) is a neurological disease characterized by headache and recurrent vertigo, usually accompanied by dizziness, nausea, vomiting and balance problem. The lifetime prevalence of VM is between 1% and 3% in the general population [1, 2], and it is up to five times more common in women when compared to men [3]. The recurrent vertigo and migraine bring about disability, work productivity loss and superfluous health care spending, resulting in low quality of life. At present, the pathophysiology of VM remains incompletely elaborated, although genetic, epigenetic, and environmental factors are probably important for its development. Most theories concerned with the pathogenesis of VM based on knowledge of migraine itself. It is proposed that the central mechanism of VM may be explained by interactions between vestibular and pain pathways [4, 5].

In the past decade, neuroimaging studies especially those using functional magnetic resonance imaging (fMRI) have greatly improved our understanding of the neural basis of VM. In a task-based fMRI study using caloric vestibular stimulation, Russo et al. detected enhanced thalamus activation in VM patients relative to both healthy controls (HC) and patients with migraine without aura (MWoA), suggesting a vestibulo-thalamo-cortical dysfunction [6]. Compared with task-based fMRI, resting-state fMRI serves as an imaging technique that could examine brain function independent of stimulus or tasks [7]. Therefore, it is easier to conduct and has been increasingly adopted in recent VM researches. The analytic approaches for resting-state fMRI data can be classified in two categories, one addressing functional connectivity (FC) and the other one addressing local activity. Common measures in the latter category include amplitude of low frequency fluctuations (ALFF) and regional homogeneity (ReHo). In a resting-state fMRI study, Li et al. found both increased ALFF and ReHo in the right temporal lobe in VM patients relative to HC [8]. Two researches on VM investigated ALFF and then evaluated FC of regions with group difference in ALFF. One revealed decreased and increased ALFF in the putamen and lingual gyrus, respectively, as well as increased putamen FC with cerebellum and decreased putamen FC with cingulate/paracingulate cortex in VM compared with both MWoA patients and HC [9]. The other one reported decreased ALFF in the thalamus, which presented decreased FC with medial prefrontal cortex and temporal lobe in the VM versus HC comparison [10]. By examining FC using a seed-based approach, Chen et al. showed abnormal functional coupling with the thalamus and parietal operculum cortex in VM patients relative to HC [11, 12]. Furthermore, atypical FC within or between resting-state networks, including the visual, auditory, sensorimotor network, as well as salience, executive and default mode network has been observed in resting-state fMRI studies on VM [13–15].

It is apparent that resting-state fMRI studies on VM often yield discrepant findings. The inconsistent and even contradictory results may be partially explained by the small sample size, clinical heterogeneity of patients and different analytic methods in prior literature. Moreover, almost all previous studies related to VM took the entire time series as a whole when calculating resting-state fMRI indices such as ALFF, Reho, and FC. However, it has now been proved that the human brain function is highly active and changeable over time [16]. Therefore, static indices of resting-state fMRI could not capture the temporal property of intrinsic brain activity (iBA). In this regard, the dynamic indices could provide complementary information and may be used to better characterize and differentiate brain conditions. Indeed, studies of the dynamic FC of iBA using the sliding time window method have contributed to the uncovering of neural mechanisms of multiple neurological disease, including subjective cognitive decline [17], depression [18, 19], and Parkinson diseases [20]. To date, only one VM study explored the time-varying attributes of iBA, and discovered atypical dynamic FC between resting-state networks in patients with VM when compared with HC [21]. However, to our knowledge, no prior studies have investigated the temporal dynamics of indices that reflect regional brain function. Additionally, concordance for iBA measures, which could capture phenotypic traits and relates to disease states [22], remains to be determined in VM research field.

Therefore, in this resting-state fMRI study, we examined the temporal variation of two widely applied “local” iBA indices, including ALFF and ReHo, in VM patients and HC using sliding time-window analysis with a relatively large sample size. Within-subject concordance of these two measures was also explored and compared between the two groups. We hypothesized that the VM patients may exhibit abnormal dynamics of ALFF and ReHo in regions related to vestibular processing, such as the temporoparietal cortical areas, and brain functional changes in several brain regions may correlate with clinical measures.

## Materials and methods

### Subjects and clinical assessment

The present study was approved by the ethics committee of the First Affiliated Hospital of Soochow University and accorded with the Declaration of Helsinki. Written informed consents were achieved from all subjects prior to their participation. Sixty-seven right-handed patients with VM were recruited from the vertigo and migraine outpatient center between October 2020 and December 2022. VM was diagnosed according to the criteria published by the Bárány Society and International Headache Society (ICHD-3 beta, appendix) [23, 24]. Videonystagmography, vestibular caloric test, video head impulse and audiometry tests were performed to rule out peripheral vestibular diseases. Demographic and clinical data were collected with a standardized questionnaire, including gender, age, education level, migraine disease duration, vertigo disease duration, headache frequency (days per month), a 10-point Visual Analog Scale (VAS), Dizziness Handicap Inventory (DHI), Migraine Disability Assessment Scale (MIDAS), Headache Impact Test-6 (HIT-6), Patient Health Questionnaire-9 (PHQ-9) and Generalized Anxiety Disorder-7 (GAD-7).

All included VM patients were not under regular preventive therapy and had not taken any therapeutic drugs within 3 days before the imaging evaluation. We managed to conduct the MRI scanning for the VM patients during interictal periods. Patients were considered to be in interictal state if they were free of migraine and vertigo attack at least 3 days before and 1 days after the MRI acquisition. The HC group consisted of 95 healthy individuals with matched gender, age and educational level. Subjects in the HC group should not have a personal or family history of migraine, vertigo, and any other types of primary headache. Exclusion criteria for all participants were as follows: left-handedness; age < 18years or > 65 years; other neurological, psychiatric disorder; cardiovascular or metabolic disease; drug or alcohol abuse; MRI contraindications; and excessive movement during the MRI scanning (head translation > 1.5 mm or rotation > 1.5º at any direction).

### MRI acquisition

We collected imaging data with a 3.0 Tesla scanning system (MAGNETOM Skyra, Siemens Healthcare, Erlangen, Germany) with a 16-channel head and neck joint coil. Foam padding and earplugs were used to minimize head movement. Sagittal fast spoiled gradient recalled echo sequence was used to acquire high resolution T1-weighted anatomic images: TR = 2300 ms, TE= 2.98 ms, matrix = 256 × 256, FOV = 256 × 256 mm^2^, slice thickness = 1 mm, slice number = 192. The structural images were checked by two experienced radiologists to exclude the visible lesions. Resting-state fMRI images were collected using EPI sequence with the following parameters: TR = 2000 ms, TE = 30 ms, flip angle = 90◦, matrix = 64 × 64, FOV = 256 × 256 mm^2^, slice thickness = 4 mm, slice number = 240. All subjects were instructed to relax with their eyes closed, stay awake but do not think about anything in particular during the functional imaging.

### Image preprocessing

The acquired functional data need to undergo significant preprocessing steps prior to analysis for they consisted of changes induced by neuronal activation, as well as non-neuronal nuisance fluctuations that are mainly due to participant motion artefacts, respiration, cardiac action and scanner artefacts (e.g. drifts). The image preprocessing was performed using a toolbox named Data Processing Assistant for Resting-State fMRI (DPARSF; http://rfmri.org/DPARSF). First, format of imaging data is converted from DICOM to NIFTI. For functional images, the first 10 time points of data were deleted to reduce the influence of initial instable magnetization and allow subjects to adapt to the environment. The remaining 230 volumes underwent slice time correcting, realignment, and co-registering with the high resolution T1-weighted images. The co-registered structural images were then segmented into gray matter, white matter (WM), and cerebrospinal fluid (CSF), and spatially normalized into the standard Montreal Neurological Institute (MNI) space with a final size of 3 × 3 × 3 mm^3^. The acquired normalization matrix was then applied to the functional data. Additionally, removal of linear trends and band-pass filtering (0.01–0.08 Hz) were employed to reduce the low-frequency drift and high frequency physiological noise. The cut-off of 0.01-0.08 Hz is widely used because it has been shown that the fMRI signal fluctuations that most consistently produce correlations within functional networks occur within this range during resting state. For spatial smoothing of the data (to reduce bad normalization and increase signal noise ratio), we used an isotropic Gaussian kernel with 6-mm full-width half maximum. It is worth noting that spatial smoothing should be only conducted after index calculation for dynamic ReHo analysis because smoothing could influence the calculation of ReHo index. Finally, nuisance covariates, including the head motion parameters, WM signal and CSF signal were regressed to minimize the effects of non-neuronal signals. The residual time series were used for the later temporal dynamic analyses.

### Calculation of dynamics and concordance of resting-state fMRI indices

Dynamic resting-state fMRI indices were calculated by sliding time-window analysis with the software named Data Processing and Analysis of Brain Imaging (DPABI) (http://rfmri.org/DPABI). Specifically, hamming windows was used to slide the time series of resting-state fMRI to get windowed time series. For there was no consensus on the optimal choice of window size and overlap, sliding window size of 50 TR and overlap of 1 TR was firstly selected in order to optimize the balance between rapidly capturing temporal changes and reliably estimating the brain activity. Accordingly, the remaining 230 volumes of resting-state fMRI data for each individual were segmented into 181 windows totally. Within each window, we calculated the resting-state fMRI indices including ALFF and ReHo in a voxel-wise way. Standard deviation (SD) maps of ALFF and ReHo across the windows were subsequently calculated to characterize the temporal dynamic characteristics of the resting-state fMRI indices. Voxel-wise within-subject concordance between ALFF and ReHo was generated by calculating the Kendall’s W for ALFF and ReHo across the time windows for each voxel. After that, Z-standardization was applied to these maps across all voxels within the group mask to reduce the global activities variability effects among subjects. As mentioned above, the dynamic maps of Reho were smoothed at this time with a 6-mm FWHM Gaussian kernel. Furthermore, the volume-wise within-subject concordance between indices was also generated by calculating the Kendall’s W for ALFF and ReHo across all voxels for each time window. To evaluate the stability of sliding time-window analysis, we also calculated the dynamic indices with other window sizes (30 and 70 TRs).

### Statistics

The SPSS 22.0 (Chicago, IL, USA) software was utilized to detect group differences in the demographic data, clinical characteristics. To be specific, gender distribution was compared with Chi-squared test. The continuous data, such as age, education level, were analyzed using independent t tests. The metrics (mean and SD across time windows) of volume-wise concordance between ALFF and ReHo were also compared by using independent t tests, with age, gender, and education level as covariates. The significance threshold was set as P < 0.05.

Analysis of dynamic resting-state fMRI indices was performed using the DPABI toolbox. Independent t test was conducted in a voxel-wise way to compare standardized dynamic ALFF, dynamic ReHo and within-subject temporal concordance maps between the two groups with the overlapping part of VM and HC group masks as the final mask. The group mask for the VM group and the HC group was generated, respectively, using DPABI software by setting the percentage to assure that the 90% of subjects have the voxels of the group mask in their EPI automask. Age, gender, education level and motion parameter were included as covariates and removed by regression during statistical analysis in order to reduce confounding influence on the result. We performed multiple comparison correction on the basis of Gaussian random field (GRF) theory within the final mask mentioned above, with a voxel-level *P* < 0.005 and cluster-level *P* < 0.05. For the data using a window size of 50 TRs, the corresponding cluster size threshold for analyses of dynamic ALFF, dynamic ReHo and within-subject temporal concordance was 161, 82, and 67 voxels, respectively.

The dynamics indices and within-subject temporal concordance in regions with significant group differences were extracted, and partial correlation analyses were utilized to explore their association with the clinical characteristics (including the migraine and vertigo disease duration, headache frequency, VAS and DHI) across all VM patients, controlling for age, gender, education level, and motion parameter. Partial correlation analyses were also performed between the clinical characteristics and metrics of volume-wise concordance in the VM group. For the data using a window size of 50 TRs, correlation analyses were multiple comparison corrected with Bonferroni correction. Specifically, the number of correlation analyses for dynamics indices and within-subject temporal concordance is 35 (5×7; 5 clinical variables of concern, and 7 identified clusters) and the number of correlation analyses for volume-wise concordance is 10 (5×2; 5 clinical variables, and 2 metrics of volume-wise concordance). Accordingly, *P* < 0.05/45 was considered to be significant for correlation analyses. For the data using a window size of 30 and 70 TRs, we only focused on stability of the correlation result that was identified by analyzing data using a window size of 50 TRs, therefore *P* < 0.05 was considered to be significant for these correlation analyses.

To explore the gender specificity of our results, the analyses mentioned above were additionally performed in the female population. Specifically, clinical characteristics and brain imaging measures were compared between the female VM subgroup and the female HC subgroup. For brain imaging data, multiple comparison correction was performed on the basis of Gaussian random field (GRF) theory, with a voxel-level *P* < 0.005 and cluster-level *P* < 0.05. We also investigated the relationship between clinical indices and brain imaging measures using the same method for the whole population as mentioned above, with a focus on the stability of the correlation result identified by analyzing data of the whole population. Therefore, *P* < 0.05 was considered to be significant for the correlation analyses.

## Results

### Demographic data and clinical characteristics

Table 1 showed the demographic and clinical data for the patient and control groups. In this study, six VM patients and seven HCs were excluded from further analyses because of excessive head motion during the image acquisition. Four additional VM patients were excluded because of the ischemic lesions. Therefore, 57 VM patients and 88 control subjects were included in the final analyses. There were no significant differences in age (*P* = 0.425), gender distribution (*P* = 0.342), and years of education (*P* = 0.202) between the two groups.

**Table 1:** Demographic data and clinical characteristics of subjects

	VM group	HC group	*p* value
Age (years)	46.44±12.24	44.69±13.20	0.425^a^
Gender (male/female)	7/50	16/72	0.342^b^
Education level (years)	10.47±4.86	11.59±5.30	0.202^a^
migraine disease duration (years)	11.97±11.46		
vertigo disease duration (years)	7.24±8.41		
Headache frequency/month	2.97±2.93		
VAS	6.57±1.74		
DHI	51.29±17.23		
MIDAS	13.34±11.95		
HIT-6	55.85±10.75		
PHQ-9	5.88±5.48		
GAD-7	4.75±4.48		

*VM* Vestibular migraine, *HC* Healthy control, *VAS* Visual Analog Scale, *DHI* Dizziness Handicap Inventory, *MIDAS* Migraine Disability Assessment Questionnaire, *HIT-6* Head ache Impact Test-6, *PHQ* Patient Health Questionnaire, *GAD* Generalized Anxiety Disorder

^a^*p* value obtained with independent *t* test

^b^*p* value obtained with chi-square test

### Dynamics and concordance of resting-state fMRI indices

**Fig. 1:**
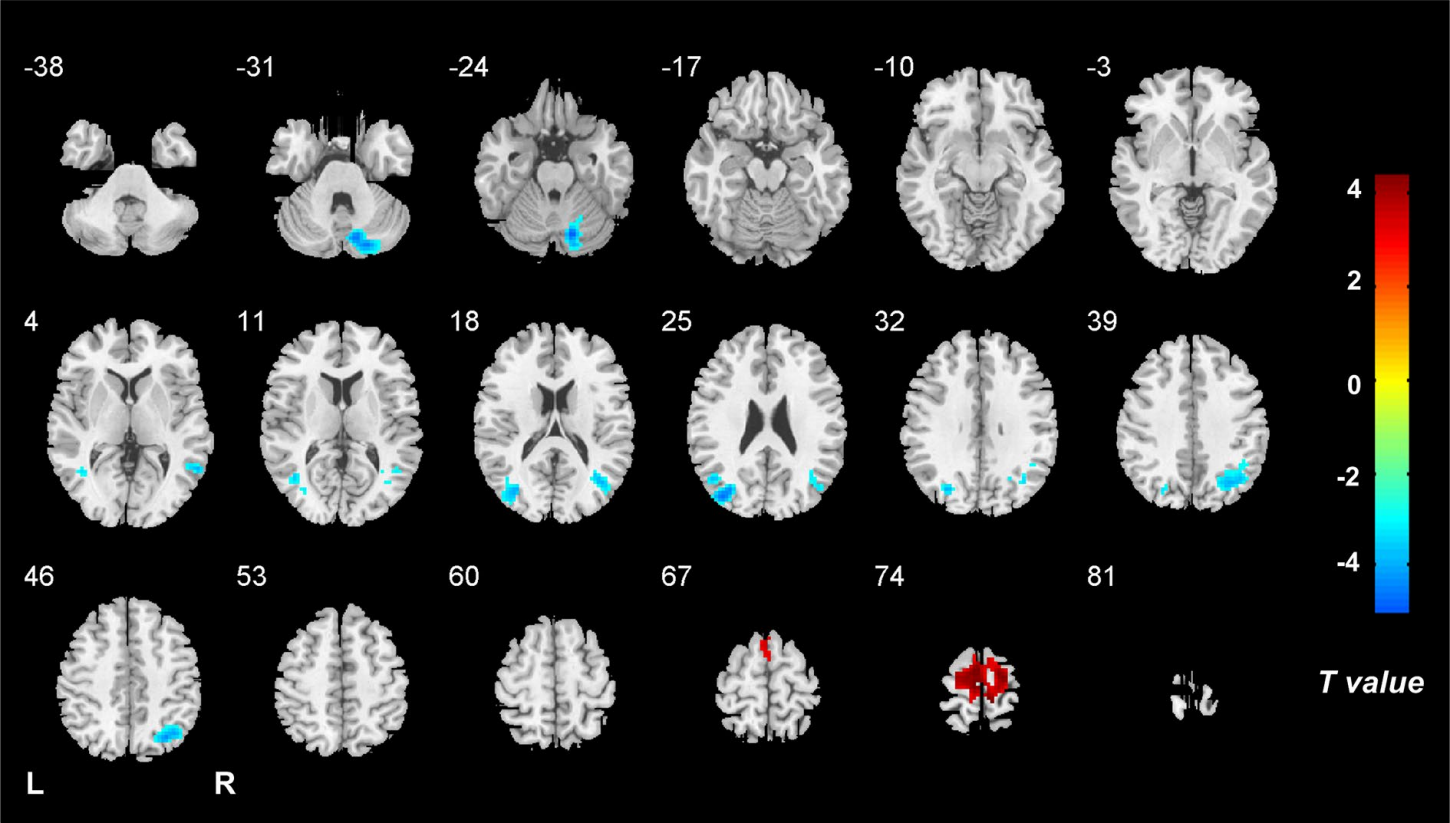
Brain regions with altered dynamics of ALFF in VM. Compared with HCs, patients with VM show significantly increased ALFF dynamics in the bilateral paracentral lobule, supplementary motor area and precentral gyrus, as well as decreased dynamic ALFF in the right cerebellum posterior lobe, bilateral angular gyrus, middle/superior occipital gyrus and middle temporal gyrus. Note: ALFF, amplitude of low-frequency fluctuations; VM, vestibular migraine; HCs, healthy controls

**Fig. 2:**
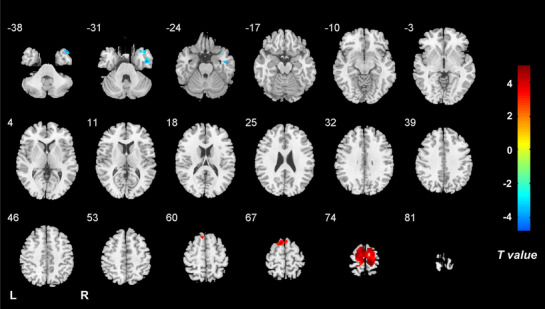
Brain regions with altered dynamics of ReHo in VM. Compared with healthy controls, patients with VM show significantly increased ReHo dynamics in the bilateral paracentral lobule, supplementary motor area and precentral gyrus, and decreased ReHo dynamics in the right temporal pole/inferior temporal gyrus. Note: ReHo, regional homogeneity; VM, vestibular migraine; HCs, healthy controls

**Fig. 3:**
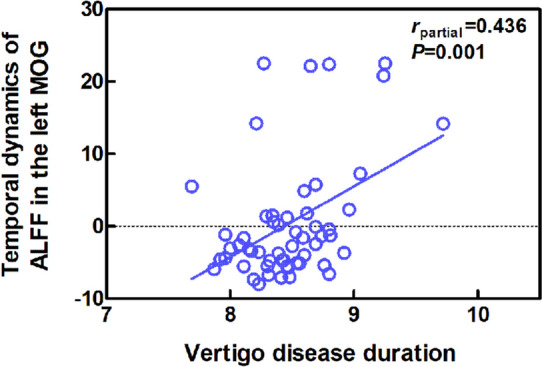
Correlation of vertigo disease duration with ALFF dynamics (Z score standardized) of the left MOG in the VM group. Note: ALFF, amplitude of low-frequency fluctuations; VM, vestibular migraine; MOG, middle occipital gyrus

Compared with the HC group, the VM group showed significantly increased ALFF dynamics in the bilateral paracentral lobule (PCL), supplementary motor area (SMA) and precentral gyrus (PreCG), as well as decreased dynamic ALFF in the right cerebellum posterior lobe, bilateral angular gyrus, middle occipital gyrus (MOG)/superior occipital gyrus and middle temporal gyrus (MTG) (Table 2, Fig. 1). The intergroup comparison also revealed increased ReHo dynamics in the bilateral PCL, SMA and PreCG, and decreased ReHo dynamics in the right temporal pole (TP)/inferior temporal gyrus in the VM patients relative to controls (Table 2, Fig. 2). Moreover, a positive correlation was observed between ALFF dynamics in the left MOG and vertigo disease duration across all VM patients (partial correlation = 0.436, *P* = 0.001, after excluding the outlier and influence point) (Fig. 3). With regard to temporal dynamics ALFF and ReHo, the results acquired using a window size of 30 or 70 TR were similar to those obtained with a window size of 50 TR (Additional files 1 and 2).

**Table 2:** Difference in dynamics and voxel-wise concordance of ALFF and ReHo between VM patients and healthy controls

Brain regions	Hemi	Cluster size (voxels)	MNI coordinate (x, y, z)	Peak* t* value
**Dynamics of ALFF**				
PCL/SMA /PreCG	L/R	287	-6,-21,75	4.39
Cerebellum posterior lobe	R	184	12,-72,-27	-4.94
Angular gyrus/MOG /MTG	L	207	-39,-78,21	-4.75
Angular gyrus/MOG /MTG	R	293	30,-66,45	-4.36
**Dynamics of ReHo**				
PCL/SMA /PreCG	L/R	454	9,-33,75	5
TP/ITG	R	86	42,18,-36	-4.73
**Voxel-wise concordance**				
PCL/SMA /PreCG	L/R	228	9,-6,75	4.76

*ALFF* Amplitude of low-frequency fluctuations, *ReHo* Regional homogeneity, *VM* vestibular migraine, *PCL* Paracentral lobule, *SMA* Supplementary motor area, PreCG Precentral gyrus, *MOG* Middle occipital gyrus, *MTG* Middle temporal gyrus, *TP* temporal pole, *ITG* Inferior temporal gyrus

**Fig. 4:**
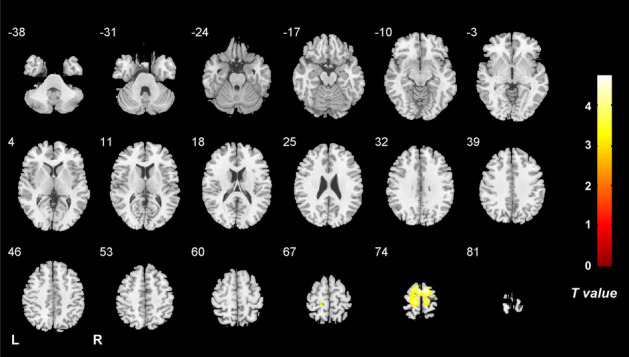
Brain regions with altered voxel-wise temporal concordance between ALFF and ReHo in VM. Compared with healthy controls, patients with VM show significantly increased voxel-wise temporal concordance between ALFF and ReHo in the bilateral paracentral lobule, supplementary motor area and precentral gyrus. Note: ALFF, amplitude of low-frequency fluctuations; ReHo, regional homogeneity; VM, vestibular migraine; HCs, healthy controls

**Fig. 5:**
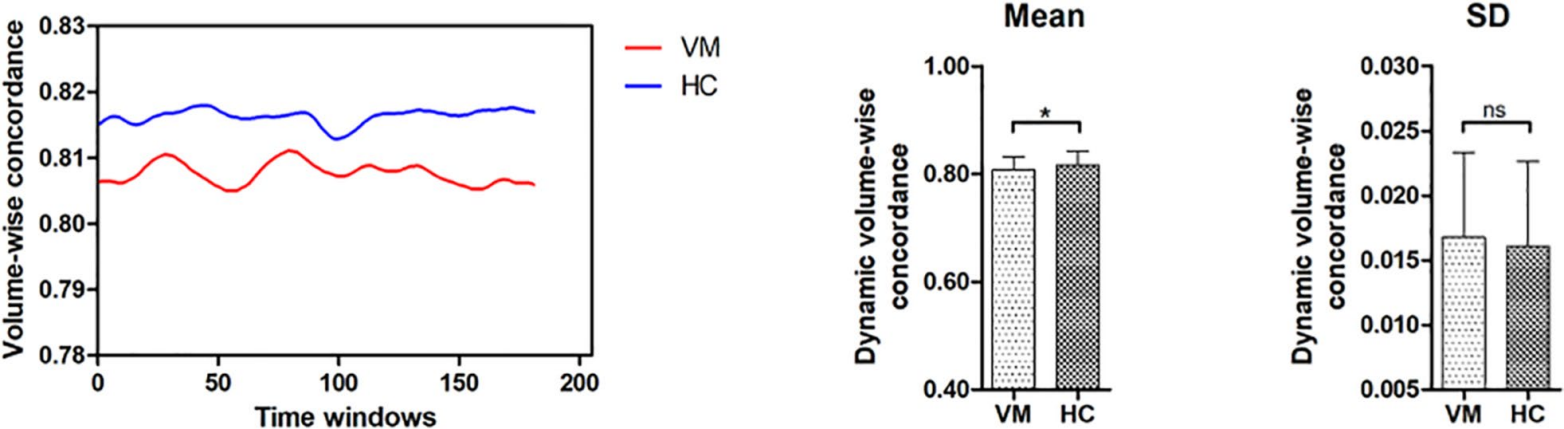
Comparison of volume-wise concordance indices between the VM and HC groups. **A** Time series of volume-wise concordance between ALFF and ReHo for typical subjects in the VM group and HC group. **B** Group comparison of the mean of volume-wise concordance between ALFF and ReHo. **C** Group comparison of the SD of volume-wise concordance between ALFF and ReHo. Note: VM, vestibular migraine; HC, healthy controls; ALFF, amplitude of low-frequency fluctuations; ReHo, regional homogeneity; SD, Standard deviation. *P < 0.05; ns, not significant

Comparing the voxel-wise concordance maps between the two groups, significantly increased concordance of ALFF and ReHo in the bilateral PCL, SMA and PreCG was found in the VM group relative to the HC group (Table 2, Fig. 4). T. With regard to the volume-wise concordance, the VM group demonstrated greater mean value of concordance between ALFF and ReHo when compared with the HC group (*P* = 0.029) (Table 3, Fig. 5). There was no significant statistical difference in the standard deviation of the volume-wise concordance between ALFF and ReHo in the VM versus HC comparison (*P* = 0.620) (Table 3, Fig. 5). The clinical measures showed no significant correlation with the voxel-wise concordance or metrics of the volume-wise concordance between ALFF and ReHo. With respect to concordance between ALFF and ReHo, the results obtained with a window size of 30 or 70 TR were similar to those achieved using a window size of 50 TR (Additional files 1 and 2).

**Table 3:** Comparison of volume-wise concordance indices between the VM group and the HC group

	VM	HC	*p* value
Mean	0.808±0.024	0.816±0.026	0.029
SD	0.017±0.007	0.016±0.007	0.620

*VM* Vestibular migraine, *HC* Healthy control, *SD* Standard deviation

### Analyses for the female population

There were no significant differences in age and years of education between the female VM subgroup and the female healthy subgroup (Additional file 3). The between-group comparison and correlation analyses of brain imaging data yielded results broadly similar to those acquired by analyzing data of subjects of both genders (Additional file 3) though some original results in the general population were not statistically significant in the female population.

## Discussion

To our knowledge, this is the first resting-state fMRI study designed to examine the temporal dynamics and concordance of regional brain function indices, including ALFF and ReHo in a relatively large sample of VM patients. As two widely used data-driven approach indicating local iBA, ALFF and ReHo analyses have been reported to have high test-retest reliability in neuroimaging research [10, 25]. Although its physiological basis is unknow, the temporal variability of iBA is believed to be functional, possibly reflecting neural adaptability to changing conditions [26] which may be damaged in neurological diseases [16–18]. The present study revealed altered dynamics of regional brain activity in the motor cortex, temporoparietal junction, temporal pole, visual areas and cerebellum, as well as increased concordance of ALFF and ReHo in the motor cortex in the VM versus HC comparison. Moreover, a positive correlation was identified between ALFF dynamics in the left MOG and vertigo disease duration of VM patients.

The results of dynamic analyses of ALFF and ReHo converged in the motor cortex, including the SMA, PCL and a small part of PreCG, which showed increased temporal variance in the VM group compared with the HC group. The findings were further supported by the observation of increased accordance between the two indices in these regions in the VM patients. The somatosensory cortex is considered as a part of multisensory areas where several sensory modalities including vestibular information converge [27]. The PCL is involved in movement of the body in the space as well as eyes movements [28]. The SMA, an area located in the dorsomedial frontal cortex, relates to not only self-initiated and triggered movements [29] but also anticipation and effective components of pain [30]. Dysfunction of this region has been reported to be associated with postural instability [31]. Therefore, abnormal dynamics and accordance in the above-mentioned regions in VM patients may indicate deficits in pain and balance control, leading to hyperreactivity, equilibrium problems and motion-induced vestibular symptoms. Partially in line with our study, Teggi et al. reported increased activation of the PCL during visual stimulation in the VM patients when compared with HCs [32]. In addition, altered resting-state FC and gray matter volume has been found for the SMA in VM in recent studies [11, 30, 33].

In the current study, the VM group showed decreased ALFF dynamics in the bilateral temporoparietal cortical areas, including angular gyrus and adjacent MTG, as well as decreased ReHo dynamics in right TP relative to the HC group. Partially consistent with our findings, previous studies have demonstrated atypical FC of the TP and temporoparietal area [10], as well as structural alteration in the inferior parietal lobule in VM patients compared with HCs [33, 34]. The temporoparietal junction is a key node of the human vestibular cortex which plays important roles in multisensory processing and is particularly sensitive for dizziness [35]. The angular gyrus is also involved in pain response and has been reported to show reduced local FC in patients with chronic migraine [36]. Interestingly, the temporoparietal junction is considered a key component of the default mode network, which subserves several cognitive processes, including episodic memory, planning, mind wandering and environmental monitoring [37]. Liao et al. found that default mode network exhibited high level temporal dynamics of intrinsic activity relative to the rest of the brain in healthy individuals [26]. The authors explained this pattern as a mechanism for efficiently response to variable stimuli. Accordingly, we speculate that our result of reduced ALFF dynamic of in the temporoparietal junction may suggest disrupted ability to adaptively response to the internal and external environment in patients with VM and relate to abnormal vestibular processing. The TP is also an associative multisensory area, assigns affective tone to pain related short-term memories and has been found to be hyper-excitable in migraine patients [38]. Therefore, decreased ReHo dynamic of this region in VM in the current study may indicate dysfunctional multisensory integration and associate with hypersensitivity to different kinds of stimuli which could be commonly found in VM sufferers.

Our data also showed decreased ALFF dynamics in the right cerebellum posterior lobe, and bilateral MOG/superior occipital gyrus that were adjacent to the posterior parietal cortex in the VM versus HC comparison. Except for motor control, the cerebellum has also been implicated in cognitive function and pain perception regulation. Hyper-metabolism in the cerebellum in VM was observed during interictal and ictal period in previous studies, which was ascribed as an adaptive mechanism to suppress the hyperactive vestibular systems [39, 40]. In accordance with our results, structural atrophy and FC alteration in this region have been reported in VM patients as well as migraine patients relative to HCs [9, 41–43]. Collectively, decreased ALFF dynamics in the cerebellum in the current study may suggest compromised ability to attenuate pain and vestibular perception. In prior research, deactivation of the occipitotemporal areas during attacks or after caloric vestibular stimulation have been observed in VM patients and HCs, respectively, suggesting a reciprocal inhibitory interaction between the visual and vestibular systems [39–41]. Regarding structural brain imaging, Messina found increased volume in the superior occipital gyrus in VM patients when compared to both HCs and migraine patients with and without aura [41]. The current study extended prior finding of abnormalities in the occipitotemporal areas to dynamics of brain activity during resting state. Given the proposed inhibitory visual-vestibular interaction, altered ALFF dynamics in this region possibly lead to disrupted vestibular processing. Intriguingly, though the VM patients had decreased ALFF dynamic, we found a positive correlation between ALFF dynamics in the left MOG and vertigo disease duration of VM patients. This may indicate a compensatory mechanism to restore visual-vestibular balance in VM patients with longer disease duration of vertigo or dizziness.

Several limitations should be pointed out in the present study. First, the power value for t test with the current sample size was estimated to be 0.83 by setting the medium effect using G*Power toolbox [44]. Though the number of subjects was relatively larger than those of the existing research, studies with larger sample size are needed to increase the statistical power. In particular, more male VM patients should be recruited to better elucidate the influence of gender on the neural mechanism of this disease. Second, the optimal fixed window width to capture the dynamics of iBA is unclear. However, the effectiveness and stability of our analysis was further proved by the similar findings acquired using a window width of 30TR and 70TR, respectively. Nevertheless, approaches independent of a rectangular sliding-window may be preferable and should be developed in the future. Third, the cross-sectional study design made it unable to infer the causal relationship between abnormal dynamics of local brain activity and VM attacks.

## Conclusion

To conclude, the present study revealed altered temporal dynamic and concordance of regional iBA indices in the motor cortex, occipital, temporoparietal cortex and cerebellum, areas that are closely related to multisensory processing, pain and vestibular control in patients with VM. Temporal dynamic analysis of regional iBA could provide complementary insight into the neuropathology of VM. ALFF dynamics in the left MOG seems to be useful biomarker for evaluating vertigo disease burden in this disorder.

### Supplementary Information

**Additional file 1:** **Supplementary Fig. 1.** Brain regions with altered dynamics of ALFF in VM patients relative to healthy controls (applied window size: 30 TR). ALFF, amplitude of low-frequency fluctuations; VM, vestibular migraine; TR, time repetition. **Supplementary Fig. 2.** Brain regions with altered dynamics of ReHo in VM patients relative to healthy controls (applied window size: 30 TR). ReHo, regional homogeneity; VM, vestibular migraine; TR, time repetition. **Supplementary Fig. 3.** Correlation of vertigo disease duration with ALFF dynamics （Z score standardized）of the left MOG in the VM group (applied window size: 30 TR). ALFF, amplitude of low-frequency fluctuations; VM, vestibular migraine; MOG, middle occipital gyrus. **Supplementary Fig. 4.** Brain regions with altered voxel-wise temporal concordance between ALFF and ReHo in VM patients relative to healthy controls (applied window size: 30 TR). ALFF, amplitude of low-frequency fluctuations; ReHo, regional homogeneity; VM, vestibular migraine. **Supplementary Fig. 5.** Comparison of volume-wise concordance indices between the VM and HC groups (applied window size: 30 TR). (A) Time series of volume-wise concordance between ALFF and ReHo for typical subjects in the VM group and HC group. (B) Group comparison of the mean of volume-wise concordance between ALFF and ReHo. (C) Group comparison of the SD of volume-wise concordance between ALFF and ReHo. VM, vestibular migraine; HC, healthy controls; ALFF, amplitude of low-frequency fluctuations; ReHo, regional homogeneity; SD, Standard deviation. *P < 0.05; ns, not significant.

**Additional file 2:** **Supplementary Fig. 6.** Brain regions with altered dynamics of ALFF in VM patients relative to healthy controls (applied window size: 70 TR). ALFF, amplitude of low-frequency fluctuations; VM, vestibular migraine; TR, time repetition. **Supplementary Fig. 7.** Brain regions with altered dynamics of ReHo in VM patients relative to healthy controls (applied window size: 70 TR). ReHo, regional homogeneity; VM, vestibular migraine; TR, time repetition. **Supplementary Fig. 8.** Correlation of vertigo disease duration with ALFF dynamics （Z score standardized）of the left MOG in the VM group (applied window size: 70 TR). ALFF, amplitude of low-frequency fluctuations; VM, vestibular migraine; MOG, middle occipital gyrus. **Supplementary Fig. 9.** Brain regions with altered voxel-wise temporal concordance between ALFF and ReHo in VM patients relative to healthy controls (applied window size: 70 TR). ALFF, amplitude of low-frequency fluctuations; ReHo, regional homogeneity; VM, vestibular migraine. **Supplementary Fig. 10.** Comparison of volume-wise concordance indices between the VM and HC groups (applied window size: 70 TR). (A) Time series of volume-wise concordance between ALFF and ReHo for typical subjects in the VM group and HC group. (B) Group comparison of the mean of volume-wise concordance between ALFF and ReHo. (C) Group comparison of the SD of volume-wise concordance between ALFF and ReHo. VM, vestibular migraine; HC, healthy controls; ALFF, amplitude of low-frequency fluctuations; ReHo, regional homogeneity; SD, Standard deviation. *P < 0.05; ns, not significant.

**Additional file 3:** **Supplementary Table 1.** Demographic data and clinical characteristics of female patients with vestibular migraine and female healthy controls. **Supplementary Fig. 11.** Brain regions with altered dynamics of ALFF in female VM patients relative to female healthy controls (applied window size: 50 TR). Multiple comparison correction is performed on the basis of Gaussian random field theory (voxel-level *P*< 0.005, cluster-level *P* < 0.05). ALFF, amplitude of low-frequency fluctuations; VM, vestibular migraine; TR, time repetition. **Supplementary Fig. 12.** Brain regions with altered dynamics of ReHo in female VM patients relative to female healthy controls (applied window size: 50 TR). Multiple comparison correction is performed on the basis of Gaussian random field theory (voxel-level *P*< 0.005, cluster-level *P* < 0.05). ReHo, regional homogeneity; VM, vestibular migraine; TR, time repetition. **Supplementary Fig. 13.** Correlation of vertigo disease duration with ALFF dynamics （Z score standardized）of the left MOG in the female VM subgroup (applied window size: 50 TR). ALFF, amplitude of low-frequency fluctuations; VM, vestibular migraine; MOG, middle occipital gyrus. **Supplementary Fig. 14.** Brain regions with altered voxel-wise temporal concordance between ALFF and ReHo in female VM patients relative to female healthy controls (applied window size: 50 TR). Multiple comparison correction is performed on the basis of Gaussian random field theory (voxel-level *P* < 0.005, cluster-level *P* < 0.05). ALFF, amplitude of low-frequency fluctuations; ReHo, regional homogeneity; VM, vestibular migraine.**Supplementary Fig. 15.** Comparison of volume-wise concordance indices between the female VM and female HC subgroups (applied window size: 50 TR). (A) Time series of volume-wise concordance between ALFF and ReHo for typical subjects in the female VM group and female HC group. (B) Group comparison of the mean of volume-wise concordance between ALFF and ReHo. (C) Group comparison of the SD of volume-wise concordance between ALFF and ReHo. VM, vestibular migraine; HC, healthy controls; ALFF, amplitude of low-frequency fluctuations; ReHo, regional homogeneity; SD, Standard deviation. *P < 0.05; ns, not significant. **Supplementary Fig. 16.** Brain regions with altered dynamics of ALFF in female VM patients relative to female healthy controls (applied window size: 30 TR). Multiple comparison correction is performed on the basis of Gaussian random field theory (voxel-level *P*< 0.005, cluster-level *P* < 0.05). ALFF, amplitude of low-frequency fluctuations; VM, vestibular migraine; TR, time repetition. **Supplementary Fig. 17.** Brain regions with altered dynamics of ReHo in female VM patients relative to female healthy controls (applied window size: 30 TR). Multiple comparison correction is performed on the basis of Gaussian random field theory (voxel-level *P*< 0.005, cluster-level *P* < 0.05). ReHo, regional homogeneity; VM, vestibular migraine; TR, time repetition. **Supplementary Fig. 18.** Correlation of vertigo disease duration with ALFF dynamics （Z score standardized）of the left MOG in the female VM subgroup (applied window size: 30 TR). ALFF, amplitude of low-frequency fluctuations; VM, vestibular migraine; MOG, middle occipital gyrus. **Supplementary Fig. 19.** Brain regions with altered voxel-wise temporal concordance between ALFF and ReHo in female VM patients relative to female healthy controls (applied window size: 30 TR). Multiple comparison correction is performed on the basis of Gaussian random field theory (voxel-level *P* < 0.005, cluster-level *P* < 0.05). ALFF, amplitude of low-frequency fluctuations; ReHo, regional homogeneity; VM, vestibular migraine.**Supplementary Fig. 20.** Comparison of volume-wise concordance indices between the female VM and female HC subgroups (applied window size: 30 TR). (A) Time series of volume-wise concordance between ALFF and ReHo for typical subjects in the female VM group and female HC group. (B) Group comparison of the mean of volume-wise concordance between ALFF and ReHo. (C) Group comparison of the SD of volume-wise concordance between ALFF and ReHo. VM, vestibular migraine; HC, healthy controls; ALFF, amplitude of low-frequency fluctuations; ReHo, regional homogeneity; SD, Standard deviation. *P < 0.05; ns, not significant. **Supplementary Fig. 21.** Brain regions with altered dynamics of ALFF in female VM patients relative to female healthy controls (applied window size: 70 TR). Multiple comparison correction is performed on the basis of Gaussian random field theory (voxel-level *P*< 0.005, cluster-level *P* < 0.05). ALFF, amplitude of low-frequency fluctuations; VM, vestibular migraine; TR, time repetition. **Supplementary Fig. 22.** Brain regions with altered dynamics of ReHo in female VM patients relative to female healthy controls (applied window size: 70 TR). Multiple comparison correction is performed on the basis of Gaussian random field theory (voxel-level *P*< 0.005, cluster-level *P* < 0.05). ReHo, regional homogeneity; VM, vestibular migraine; TR, time repetition. **Supplementary Fig. 23.** Correlation of vertigo disease duration with ALFF dynamics （Z score standardized）of the left MOG in the female VM subgroup (applied window size: 70 TR). ALFF, amplitude of low-frequency fluctuations; VM, vestibular migraine; MOG, middle occipital gyrus. **Supplementary Fig. 24.** Brain regions with altered voxel-wise temporal concordance between ALFF and ReHo in female VM patients relative to female healthy controls (applied window size: 70 TR). Multiple comparison correction is performed on the basis of Gaussian random field theory (voxel-level *P* < 0.005, cluster-level *P* < 0.05). There is no region showing significant between-group difference in the voxel-wise temporal concordance between ALFF and ReHo. ALFF, amplitude of low-frequency fluctuations; ReHo, regional homogeneity; VM, vestibular migraine. **Supplementary Fig. 25.** Comparison of volume-wise concordance indices between the female VM and female HC subgroups (applied window size: 70 TR). (A) Time series of volume-wise concordance between ALFF and ReHo for typical subjects in the female VM group and female HC group. (B) Group comparison of the mean of volume-wise concordance between ALFF and ReHo. (C) Group comparison of the SD of volume-wise concordance between ALFF and ReHo. VM, vestibular migraine; HC, healthy controls; ALFF, amplitude of low-frequency fluctuations; ReHo, regional homogeneity; SD, Standard deviation. *P < 0.05; ns, not significant.

## Data Availability

The datasets used and/or analysed during the current study are available from the corresponding author on reasonable request.

## Abbreviations

VM: Vestibular migraine
IBA: Intrinsic brain activity
HC: Healthy controls
fMRI: Functional magnetic resonance imaging
ALFF: Amplitude of low-frequency fluctuations
ReHo: Regional homogeneity
MOG: Middle occipital gyrus
MWoA: Migraine without aura
FC: Functional connectivity
VAS: Visual Analog Scale
DHI: Dizziness Handicap Inventory
MIDAS: Migraine Disability Assessment Scale
HIT-6: Headache Impact Test-6
PHQ-9: Patient Health Questionnaire-9
GAD-7: Generalized Anxiety Disorder-7
DPARSF: Data Processing Assistant for Resting-State fMRI
WM: White matter
CSF: Cerebrospinal flui
MNI: Montreal Neurological Institute
DPABI: Data Processing and Analysis of Brain Imaging
SD: Standard deviation
GRF: Gaussian random field
PC: Paracentral lobule
SM: Supplementary motor area
PreCG: Precentral gyrus
MTG: Middle temporal gyrus
TP: Temporal pole
