# Supplementary material for: Dynamics and concordance alterations of regional brain function indices in vestibular migraine: a resting-state fMRI study
Source: J Headache Pain. 2024 Jan 5;25(1):1. doi: 10.1186/s10194-023-01705-y (PMC10768112; doi:10.1186/s10194-023-01705-y)
Supplement: Supplementary file 2 — Additional file 2: Supplementary Fig. 6. Brain regions with altered dynamics of ALFF in VM patients relative to healthy controls (applied window size: 70 TR). ALFF, amplitude of low-frequency fluctuations; VM, vestibular migraine; TR, time repetition. Supplementary Fig. 7. Brain regions with altered dynamics of ReHo in VM patients relative to healthy controls (applied window size: 70 TR). ReHo, regional homogeneity; VM, vestibular migraine; TR, time repetition. Supplementary Fig. 8. Correlation of vertigo disease duration with ALFF dynamics （Z score standardized）of the left MOG in the VM group (applied window size: 70 TR). ALFF, amplitude of low-frequency fluctuations; VM, vestibular migraine; MOG, middle occipital gyrus. Supplementary Fig. 9. Brain regions with altered voxel-wise temporal concordance between ALFF and ReHo in VM patients relative to healthy controls (applied window size: 70 TR). ALFF, amplitude of low-frequency fluctuations; ReHo, regional homogeneity; VM, vestibular migraine. Supplementary Fig. 10. Comparison of volume-wise concordance indices between the VM and HC groups (applied window size: 70 TR). (A) Time series of volume-wise concordance between ALFF and ReHo for typical subjects in the VM group and HC group. (B) Group comparison of the mean of volume-wise concordance between ALFF and ReHo. (C) Group comparison of the SD of volume-wise concordance between ALFF and ReHo. VM, vestibular migraine; HC, healthy controls; ALFF, amplitude of low-frequency fluctuations; ReHo, regional homogeneity; SD, Standard deviation. *P < 0.05; ns, not significant. [file 10194_2023_1705_MOESM2_ESM.docx]

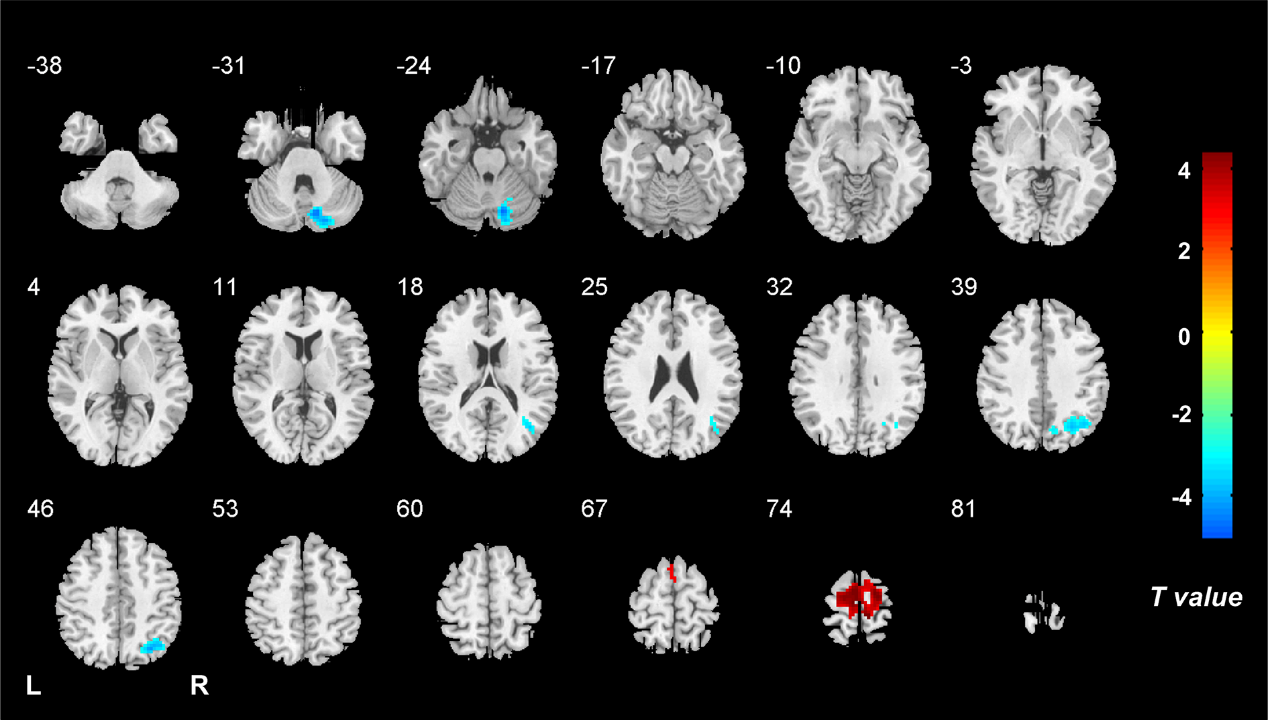


**Supplementary Fig. 6** Brain regions with altered dynamics of ALFF in VM patients relative to healthy controls (**applied window size: 70 TR**). ALFF, amplitude of low-frequency fluctuations; VM, vestibular migraine; TR, time repetition.


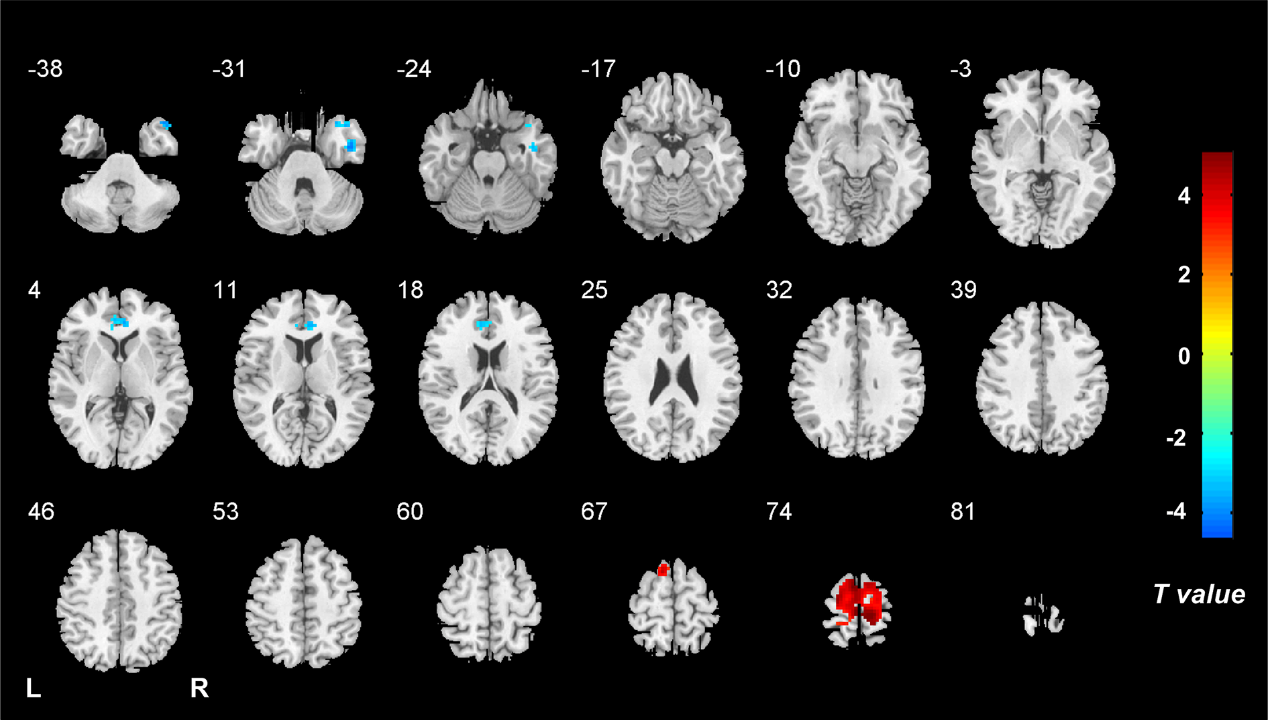


**Supplementary Fig. 7** Brain regions with altered dynamics of ReHo in VM patients relative to healthy controls (**applied window size: 70 TR**). ReHo, regional homogeneity; VM, vestibular migraine; TR, time repetition.





**Supplementary Fig. 8** Correlation of vertigo disease duration with ALFF dynamics （Z score standardized）of the left MOG in the VM group (**applied window size: 70 TR**). ALFF, amplitude of low-frequency fluctuations; VM, vestibular migraine; MOG, middle occipital gyrus.


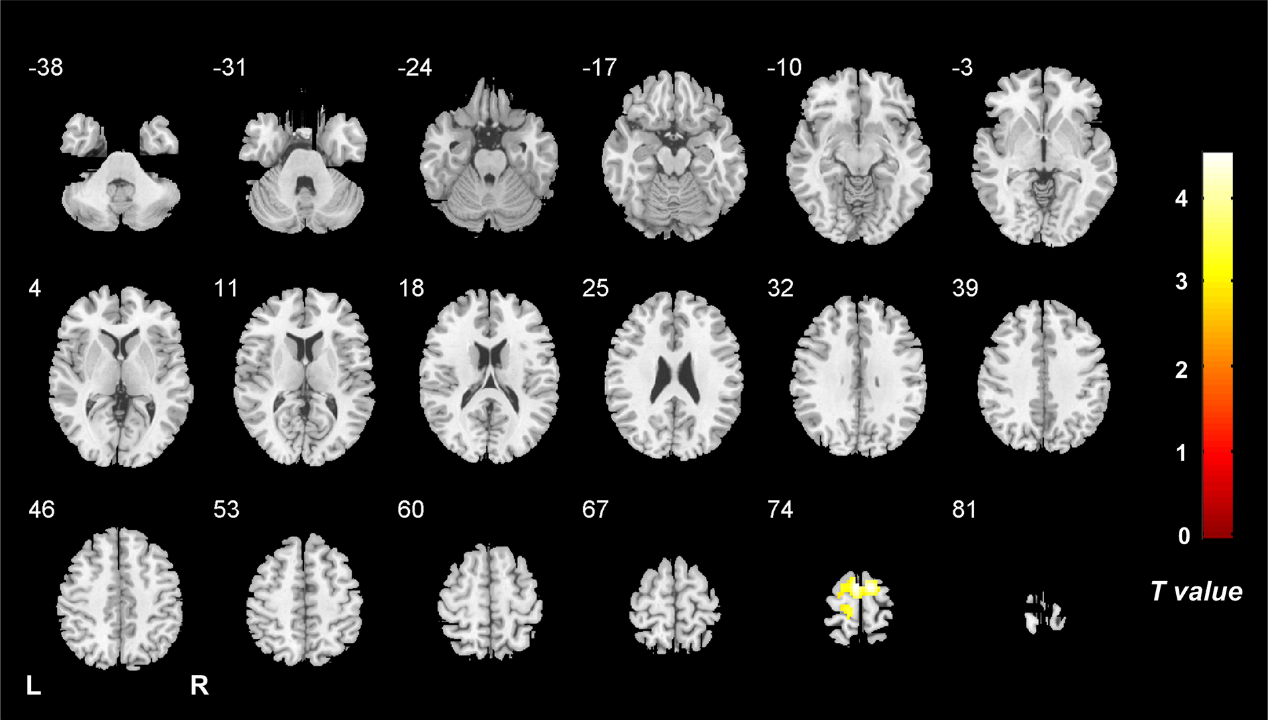


**Supplementary Fig. 9** Brain regions with altered voxel-wise temporal concordance between ALFF and ReHo in VM patients relative to healthy controls (**applied window size: 70 TR**). ALFF, amplitude of low-frequency fluctuations; ReHo, regional homogeneity; VM, vestibular migraine.


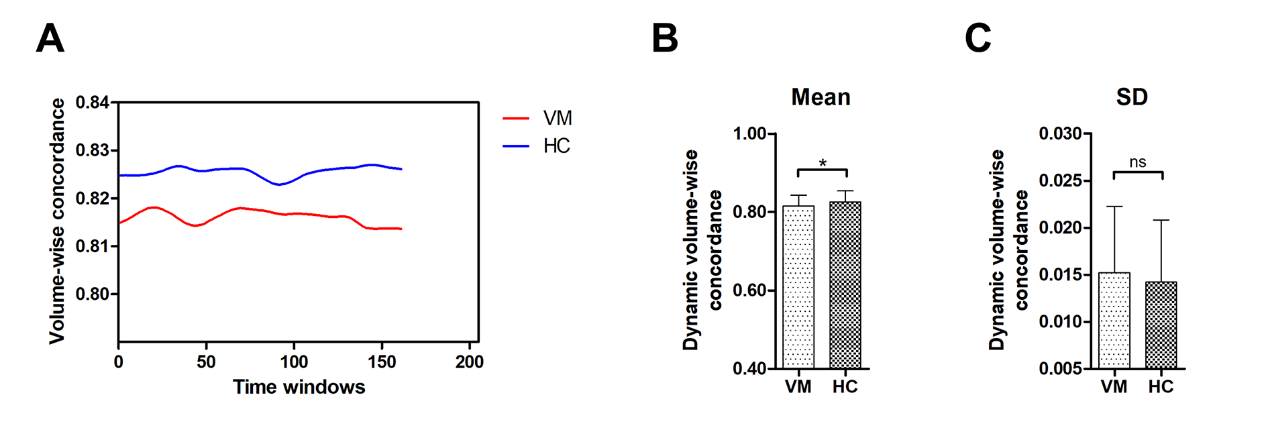


**Supplementary Fig. 10** Comparison of volume-wise concordance indices between the VM and HC groups (**applied window size: 70 TR**). (A) Time series of volume-wise concordance between ALFF and ReHo for typical subjects in the VM group and HC group. (B) Group comparison of the mean of volume-wise concordance between ALFF and ReHo. (C) Group comparison of the SD of volume-wise concordance between ALFF and ReHo. VM, vestibular migraine; HC, healthy controls; ALFF, amplitude of low-frequency fluctuations; ReHo, regional homogeneity; SD, Standard deviation. *P < 0.05; ns, not significant.
