# Supplementary material for: Dynamics and concordance alterations of regional brain function indices in vestibular migraine: a resting-state fMRI study
Source: J Headache Pain. 2024 Jan 5;25(1):1. doi: 10.1186/s10194-023-01705-y (PMC10768112; doi:10.1186/s10194-023-01705-y)
Supplement: Supplementary file 3 — Additional file 3: Supplementary Table 1. Demographic data and clinical characteristics of female patients with vestibular migraine and female healthy controls. Supplementary Fig. 11. Brain regions with altered dynamics of ALFF in female VM patients relative to female healthy controls (applied window size: 50 TR). Multiple comparison correction is performed on the basis of Gaussian random field theory (voxel-level P< 0.005, cluster-level P < 0.05). ALFF, amplitude of low-frequency fluctuations; VM, vestibular migraine; TR, time repetition. Supplementary Fig. 12. Brain regions with altered dynamics of ReHo in female VM patients relative to female healthy controls (applied window size: 50 TR). Multiple comparison correction is performed on the basis of Gaussian random field theory (voxel-level P< 0.005, cluster-level P < 0.05). ReHo, regional homogeneity; VM, vestibular migraine; TR, time repetition. Supplementary Fig. 13. Correlation of vertigo disease duration with ALFF dynamics （Z score standardized）of the left MOG in the female VM subgroup (applied window size: 50 TR). ALFF, amplitude of low-frequency fluctuations; VM, vestibular migraine; MOG, middle occipital gyrus. Supplementary Fig. 14. Brain regions with altered voxel-wise temporal concordance between ALFF and ReHo in female VM patients relative to female healthy controls (applied window size: 50 TR). Multiple comparison correction is performed on the basis of Gaussian random field theory (voxel-level P < 0.005, cluster-level P < 0.05). ALFF, amplitude of low-frequency fluctuations; ReHo, regional homogeneity; VM, vestibular migraine.Supplementary Fig. 15. Comparison of volume-wise concordance indices between the female VM and female HC subgroups (applied window size: 50 TR). (A) Time series of volume-wise concordance between ALFF and ReHo for typical subjects in the female VM group and female HC group. (B) Group comparison of the mean of volume-wise concordance between ALFF and ReHo. (C) Group comparison of [file 10194_2023_1705_MOESM3_ESM.docx]

**Supplementary Table 1** Demographic data and clinical characteristics of female patients with vestibular migraine and female healthy controls

|  | VM (N=50) | HC (N=72) | p value |
| --- | --- | --- | --- |
| Age (years) | 48.18±11.58 | 44.15±12.31 | 0.071 |
| Education level (years) | 10.02±4.67 | 11.39±5.29 | 0.143 |
| migraine disease duration (years) | 12.35±11.78 |  |  |
| vertigo disease duration (years) | 7.61±8.77 |  |  |
| Headache frequency/month | 3.08±2.88 |  |  |
| VAS | 6.74±1.74 |  |  |
| DHI | 51.66±17.29 |  |  |
| MIDAS | 13.83±11.27 |  |  |
| HIT-6 | 56.54±10.31 |  |  |
| PHQ-9 | 5.36±5.24 |  |  |
| GAD-7 | 4.46±4.28 |  |  |

Note: *p* value was obtained with independent t test. VAS, Visual Analog Scale; DHI, Dizziness Handicap Inventory; MIDAS, Migraine Disability Assessment Questionnaire; HIT-6, Headache Impact Test-6; PHQ, Patient Health Questionnaire; GAD; Generalized Anxiety Disorder.

**
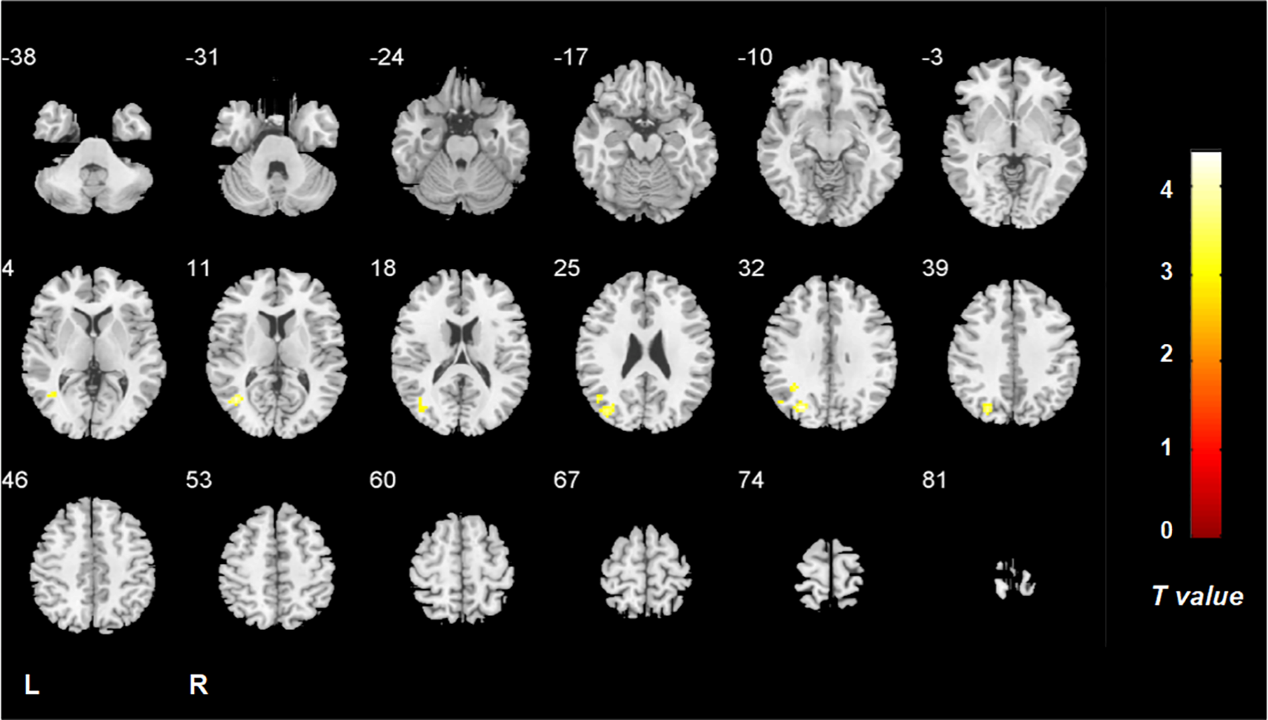
**

**Supplementary Fig. 11** Brain regions with altered dynamics of ALFF in female VM patients relative to female healthy controls (**applied window size: 50 TR**). Multiple comparison correction is performed on the basis of Gaussian random field theory (voxel-level P < 0.005, cluster-level P < 0.05). ALFF, amplitude of low-frequency fluctuations; VM, vestibular migraine; TR, time repetition.

**
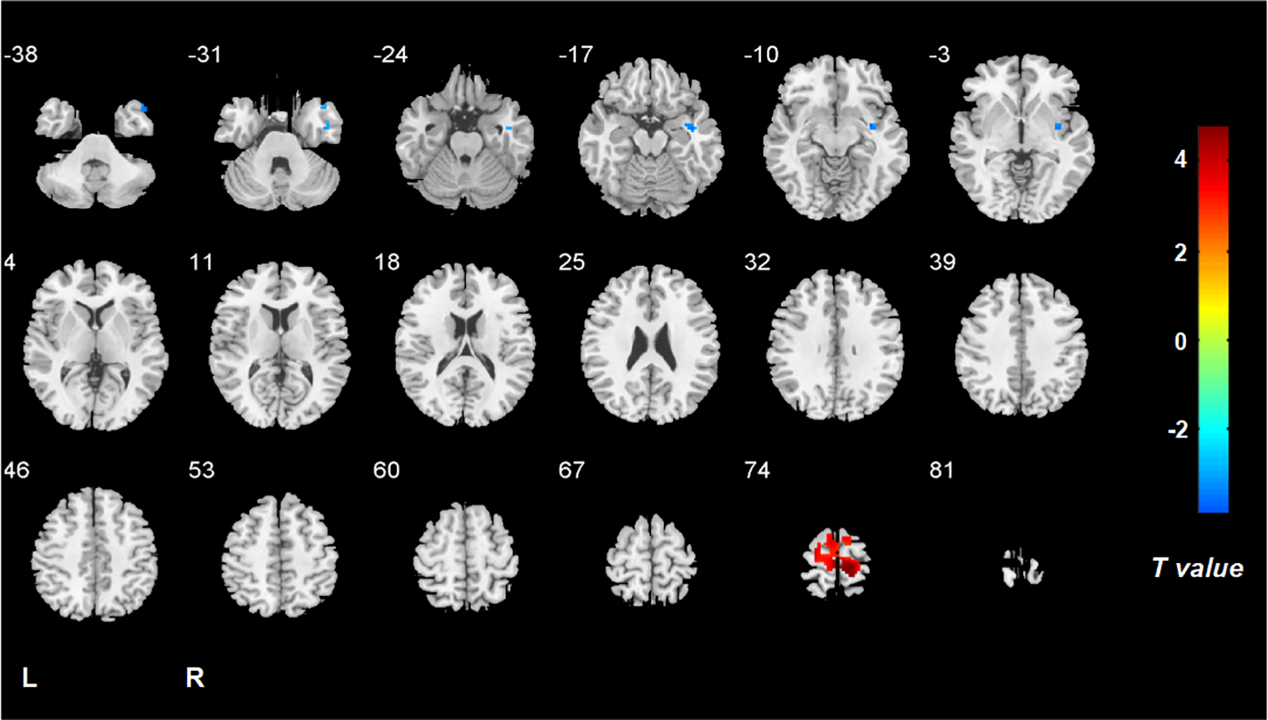
**

**Supplementary Fig. 12** Brain regions with altered dynamics of ReHo in female VM patients relative to female healthy controls (**applied window size: 50 TR**). Multiple comparison correction is performed on the basis of Gaussian random field theory (voxel-level P < 0.005, cluster-level P < 0.05). ReHo, regional homogeneity; VM, vestibular migraine; TR, time repetition.

**

**

**Supplementary Fig. 13** Correlation of vertigo disease duration with ALFF dynamics （Z score standardized）of the left MOG in the female VM subgroup (**applied window size: 50 TR**). ALFF, amplitude of low-frequency fluctuations; VM, vestibular migraine; MOG, middle occipital gyrus.

**
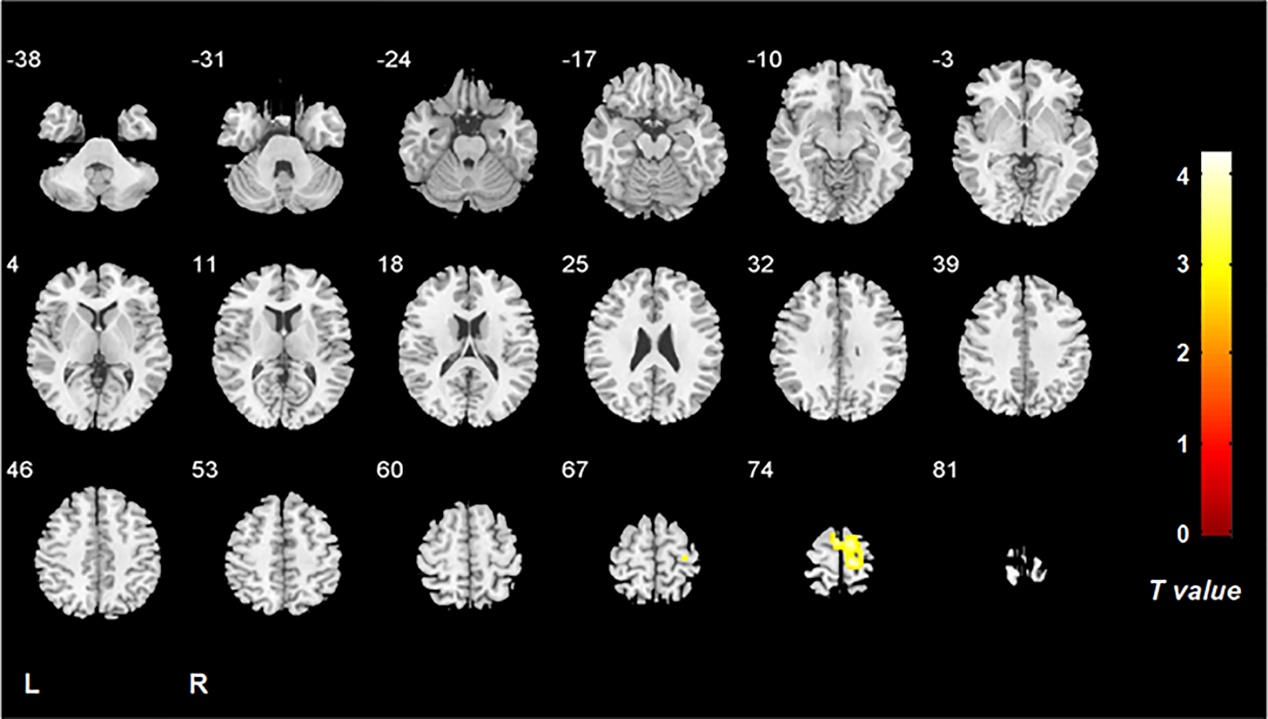
**

**Supplementary Fig. 14** Brain regions with altered voxel-wise temporal concordance between ALFF and ReHo in female VM patients relative to female healthy controls (**applied window size: 50 TR**). Multiple comparison correction is performed on the basis of Gaussian random field theory (voxel-level P < 0.005, cluster-level P < 0.05). ALFF, amplitude of low-frequency fluctuations; ReHo, regional homogeneity; VM, vestibular migraine.

**

**

**Supplementary Fig. 15** Comparison of volume-wise concordance indices between the female VM and female HC subgroups (**applied window size: 50 TR**). (A) Time series of volume-wise concordance between ALFF and ReHo for typical subjects in the female VM group and female HC group. (B) Group comparison of the mean of volume-wise concordance between ALFF and ReHo. (C) Group comparison of the SD of volume-wise concordance between ALFF and ReHo. VM, vestibular migraine; HC, healthy controls; ALFF, amplitude of low-frequency fluctuations; ReHo, regional homogeneity; SD, Standard deviation. *P < 0.05; ns, not significant.


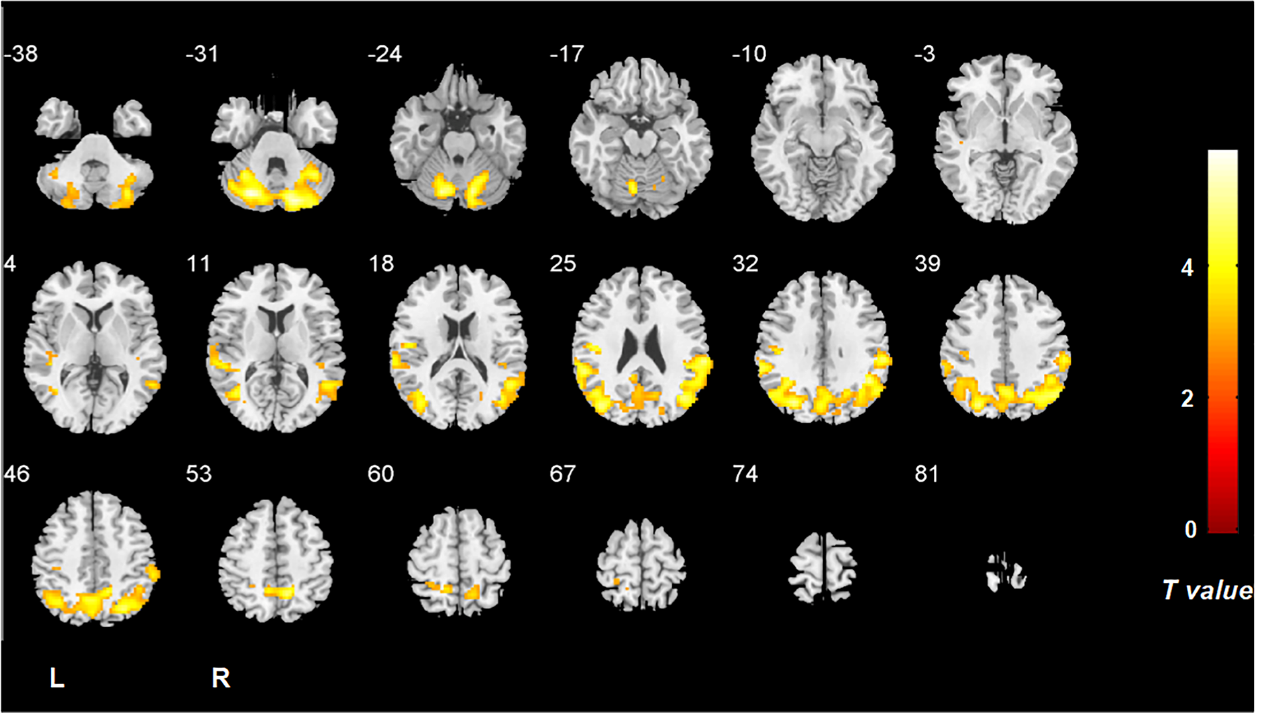


**Supplementary Fig. 16** Brain regions with altered dynamics of ALFF in female VM patients relative to female healthy controls (**applied window size: 30 TR**). Multiple comparison correction is performed on the basis of Gaussian random field theory (voxel-level P < 0.005, cluster-level P < 0.05). ALFF, amplitude of low-frequency fluctuations; VM, vestibular migraine; TR, time repetition.

**
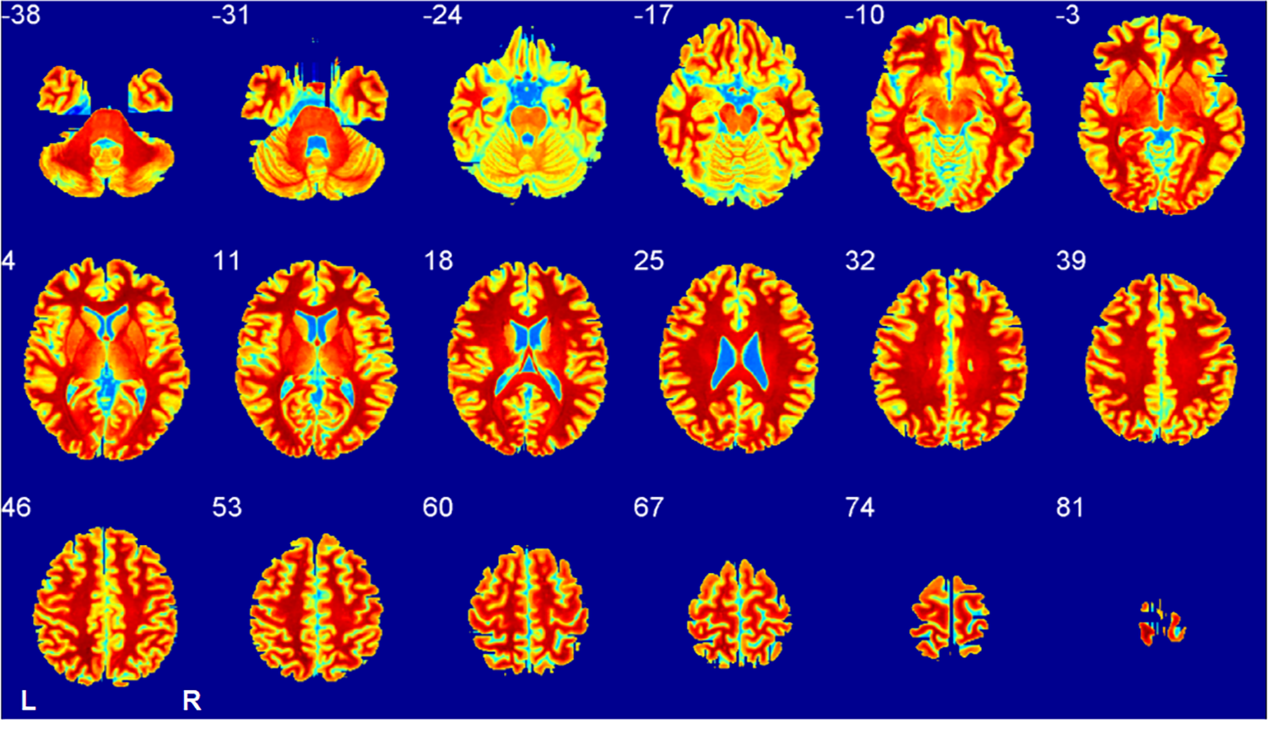
**

**Supplementary Fig. 17** Brain regions with altered dynamics of ReHo in female VM patients relative to female healthy controls (**applied window size: 30 TR**). Multiple comparison correction is performed on the basis of Gaussian random field theory (voxel-level P < 0.005, cluster-level P < 0.05). ReHo, regional homogeneity; VM, vestibular migraine; TR, time repetition.





**Supplementary Fig. 18** Correlation of vertigo disease duration with ALFF dynamics （Z score standardized）of the left MOG in the female VM subgroup (**applied window size: 30 TR**). ALFF, amplitude of low-frequency fluctuations; VM, vestibular migraine; MOG, middle occipital gyrus.


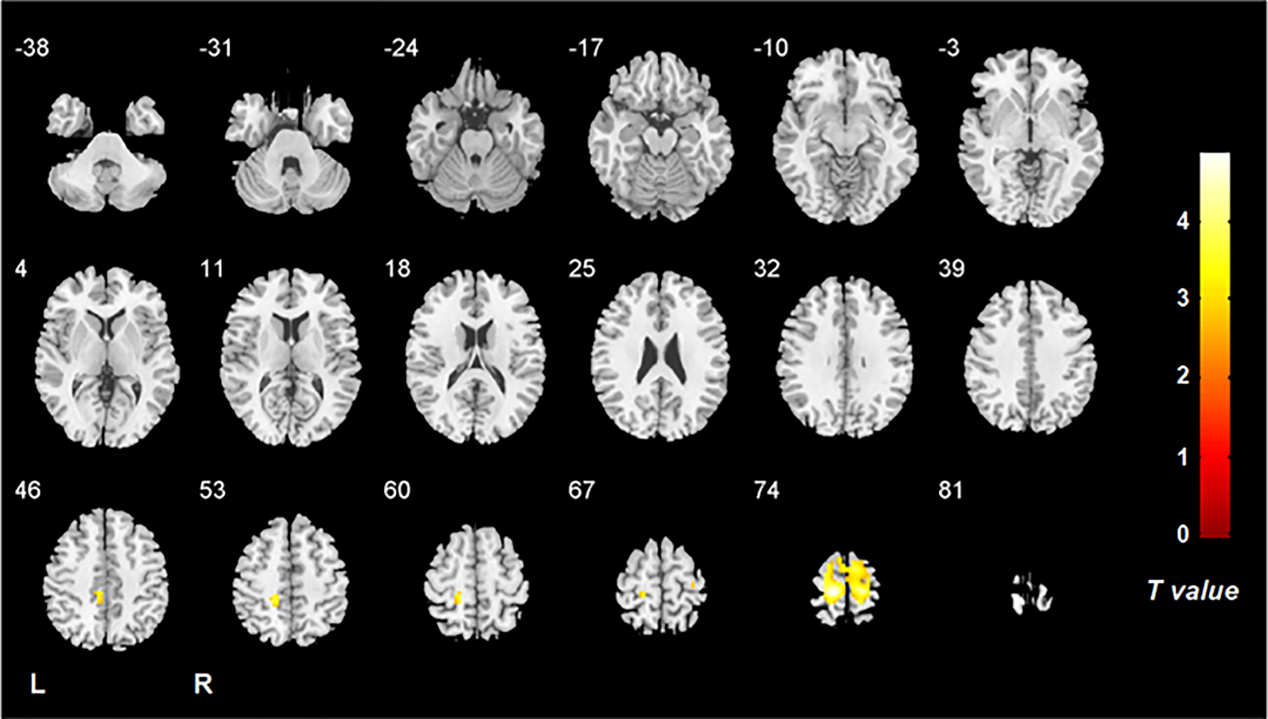


**Supplementary Fig. 19** Brain regions with altered voxel-wise temporal concordance between ALFF and ReHo in female VM patients relative to female healthy controls (**applied window size: 30 TR**). Multiple comparison correction is performed on the basis of Gaussian random field theory (voxel-level P < 0.005, cluster-level P < 0.05). ALFF, amplitude of low-frequency fluctuations; ReHo, regional homogeneity; VM, vestibular migraine.





**Supplementary Fig. 20** Comparison of volume-wise concordance indices between the female VM and female HC subgroups (**applied window size: 30 TR**). (A) Time series of volume-wise concordance between ALFF and ReHo for typical subjects in the female VM group and female HC group. (B) Group comparison of the mean of volume-wise concordance between ALFF and ReHo. (C) Group comparison of the SD of volume-wise concordance between ALFF and ReHo. VM, vestibular migraine; HC, healthy controls; ALFF, amplitude of low-frequency fluctuations; ReHo, regional homogeneity; SD, Standard deviation. *P < 0.05; ns, not significant.


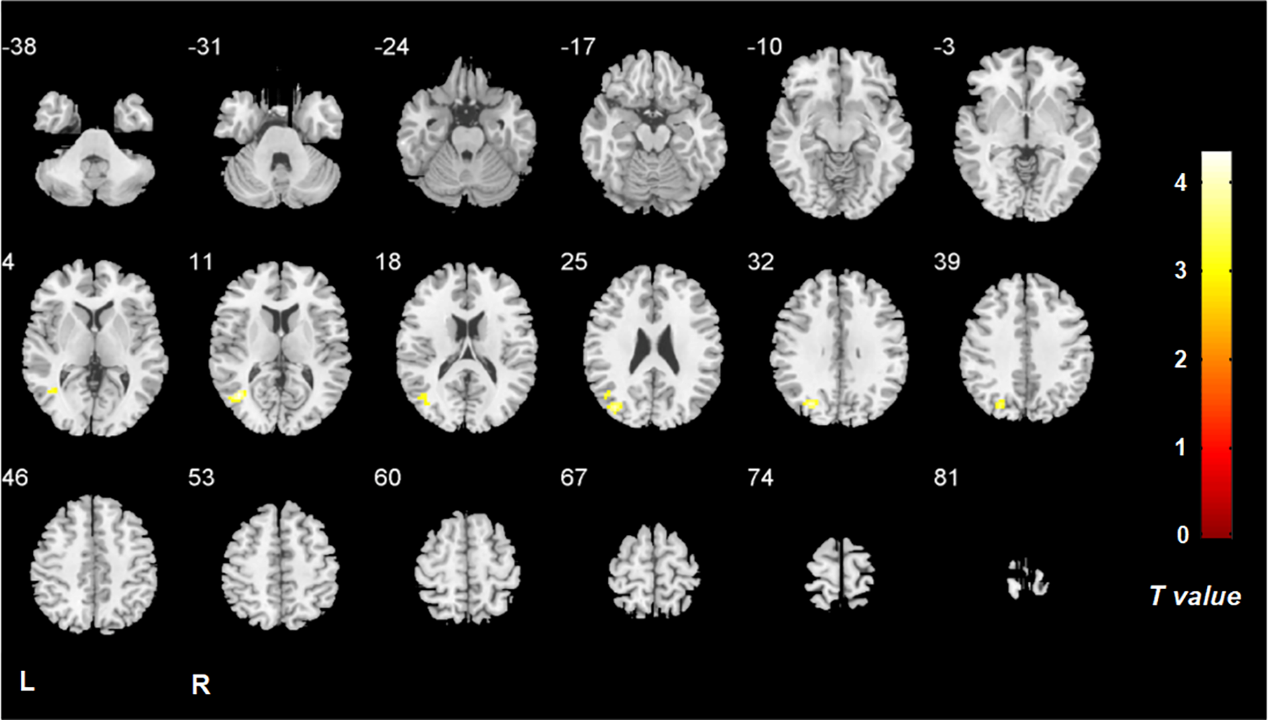


**Supplementary Fig. 21** Brain regions with altered dynamics of ALFF in female VM patients relative to female healthy controls (**applied window size: 70 TR**). Multiple comparison correction is performed on the basis of Gaussian random field theory (voxel-level P < 0.005, cluster-level P < 0.05). ALFF, amplitude of low-frequency fluctuations; VM, vestibular migraine; TR, time repetition.


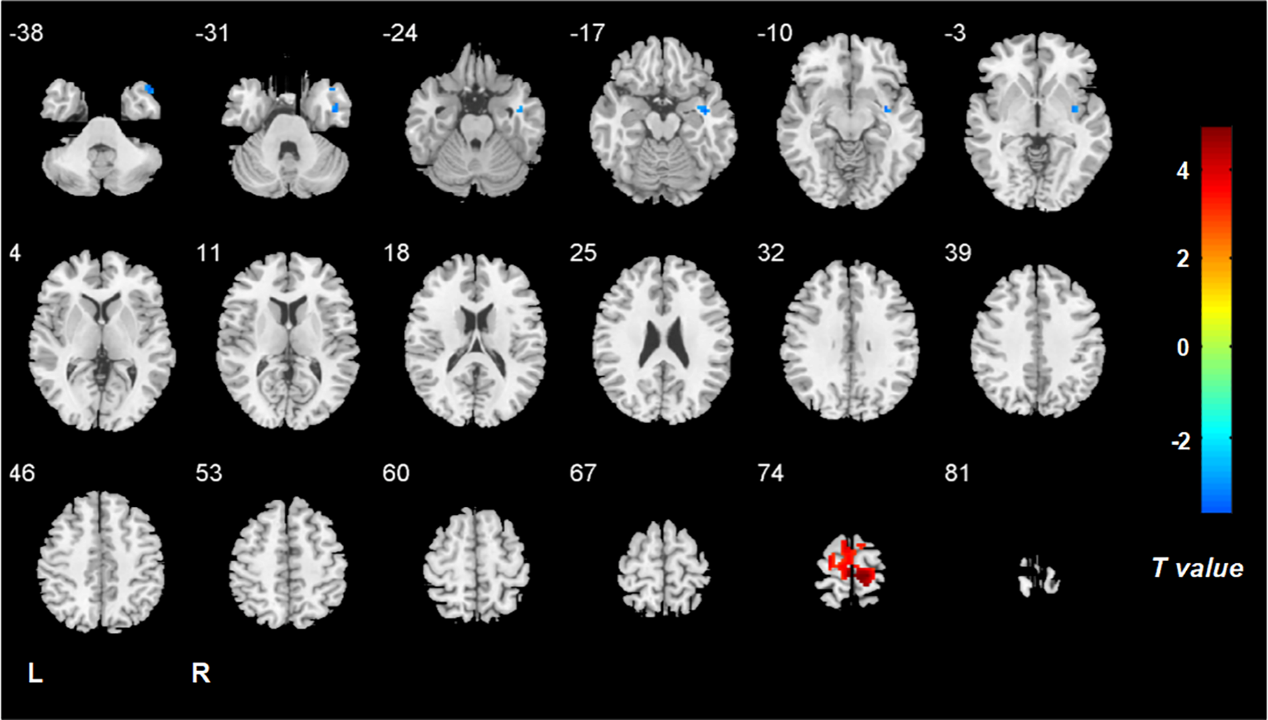


**Supplementary Fig. 22** Brain regions with altered dynamics of ReHo in female VM patients relative to female healthy controls (**applied window size: 70 TR**). Multiple comparison correction is performed on the basis of Gaussian random field theory (voxel-level P < 0.005, cluster-level P < 0.05). ReHo, regional homogeneity; VM, vestibular migraine; TR, time repetition.





**Supplementary Fig. 23** Correlation of vertigo disease duration with ALFF dynamics （Z score standardized）of the left MOG in the female VM subgroup (**applied window size: 70 TR**). ALFF, amplitude of low-frequency fluctuations; VM, vestibular migraine; MOG, middle occipital gyrus.


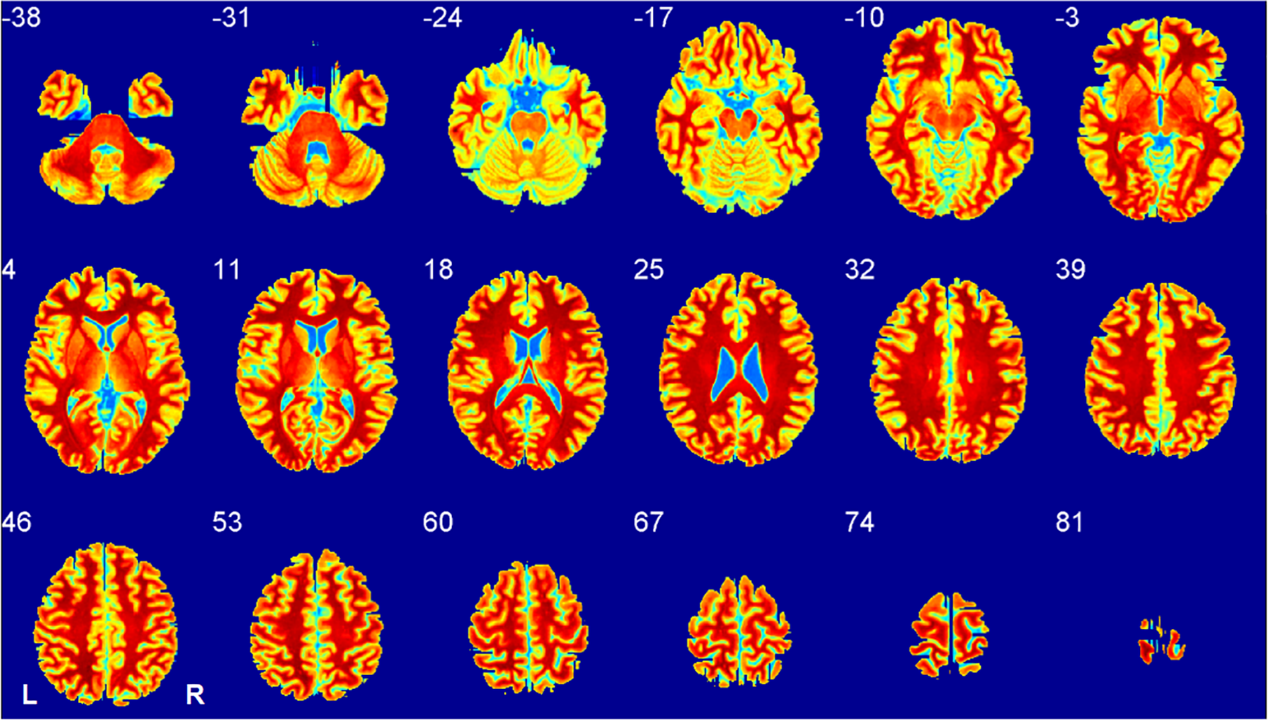


**Supplementary Fig. 24** Brain regions with altered voxel-wise temporal concordance between ALFF and ReHo in female VM patients relative to female healthy controls (**applied window size: 70 TR**). Multiple comparison correction is performed on the basis of Gaussian random field theory (voxel-level P < 0.005, cluster-level P < 0.05). There is no region showing significant between-group difference in the voxel-wise temporal concordance between ALFF and ReHo. ALFF, amplitude of low-frequency fluctuations; ReHo, regional homogeneity; VM, vestibular migraine.





**Supplementary Fig. 25** Comparison of volume-wise concordance indices between the female VM and female HC subgroups (**applied window size: 70 TR**). (A) Time series of volume-wise concordance between ALFF and ReHo for typical subjects in the female VM group and female HC group. (B) Group comparison of the mean of volume-wise concordance between ALFF and ReHo. (C) Group comparison of the SD of volume-wise concordance between ALFF and ReHo. VM, vestibular migraine; HC, healthy controls; ALFF, amplitude of low-frequency fluctuations; ReHo, regional homogeneity; SD, Standard deviation. *P < 0.05; ns, not significant.
